# Ribonucleobase Oxidation and Ribonucleases Involved in the Degradation of Oxidized RNA

**DOI:** 10.3390/biom16040564

**Published:** 2026-04-10

**Authors:** Dagoberto Grijalva-Flores, Marino J. E. Resendiz

**Affiliations:** Department of Chemistry, University of Colorado Denver, Science Building 1151 Arapahoe St., Denver, CO 80204, USA; dagoberto.grijalva-flores@ucdenver.edu

**Keywords:** RNA oxidation, oxidized RNA degradation, oxidized RNA protein binding, RNA 8-oxoG, ribonucleobase oxidation

## Abstract

Oxidation of RNA has gained interest from the community due, in part, to a link in the progression/development of disease as well as other biological processes such as apoptosis, ageing, hibernation, and signalling, amongst others. Different types of RNA with varying functions and size have been shown to be oxidized in vivo, including ribosomal RNA (rRNA), transfer RNA (tRNA), microRNA (miRNA), messenger RNA (mRNA), and mitochondrial RNA (mtRNA). This process occurs from reactions between reactive oxygen species (ROS) and all biopolymers, including RNA, from endogenous as well as exogenous sources. As a consequence, mechanisms that handle oxidized RNA are important, and enzymatic degradation is the most commonly studied process to date. This review focuses on the ribonucleases that have been shown to play a role in the degradation of oxidized RNA. While emphasis is placed on, arguably, the most common oxidatively generated chemical modification, 8-oxo-7,8-dihydroguanosine (8-oxoG), the products that arise from the oxidation of other canonical nucleosides as well as naturally occurring modifications are also discussed in the context of RNA oxidation. Processing of oxidized RNA via its enzymatic degradation is likely the main route, but a potential role of other proteins involved in the handling of oxidized RNA is hypothesized, e.g., helicases, export proteins, and extracellular environments. We postulate that this is an area with great potential for discovery.

## 1. Introduction

All biopolymers are constantly exposed to different types of stress and oftentimes undergo constant modification as a result of (or in response to) the same. Meanwhile, those occurring from reactions with reactive oxygen species (ROS) result in chemically modified molecules with important biological implications. Cellular metabolism produces ROS that are in constant flux until equilibrium is achieved [1]; however, an imbalance in these processes can lead to increased concentrations of ROS, known as oxidative stress, and with a direct impact on all kingdoms of life, including plants [2], bacteria [3], and mammals [4]. The oxidation of biomolecules occurs in important cellular constituents such as lipids, DNA, and proteins, all of which are ubiquitous and much better understood than the analogous processes in RNA. This is perhaps due to the transient nature of RNA and its inherited shorter half-life of ~60 s—20 min [5,6] compared to the other biopolymers, with some exceptions of other persistent RNA [7]. This is also coupled with the long-known mechanisms that exist for its processing/degradation [8,9]. However, the last decade has seen an increased interest and research focus on RNA oxidation [10], where its importance spans past its role in various biological mechanisms, including progression and development of disease (see Supplementary Materials, Section S1, for a comprehensive list detailing a link between RNA oxidation and such processes). RNA is also exposed to reactive nitrogen species and other alkylating agents that can ‘damage’ this biopolymer; however, those are not discussed here. This review summarizes the available literature about oxidation reactions of canonical nucleobases (more commonly studied) as well as those occurring on natural RNA modifications. Then, a transition is made to describe how oxidized RNA (containing 8-oxoG) may be handled via interactions with ribonucleases and other RNA binding proteins.

## 2. Nucleobase Oxidation

**Scheme 1:**
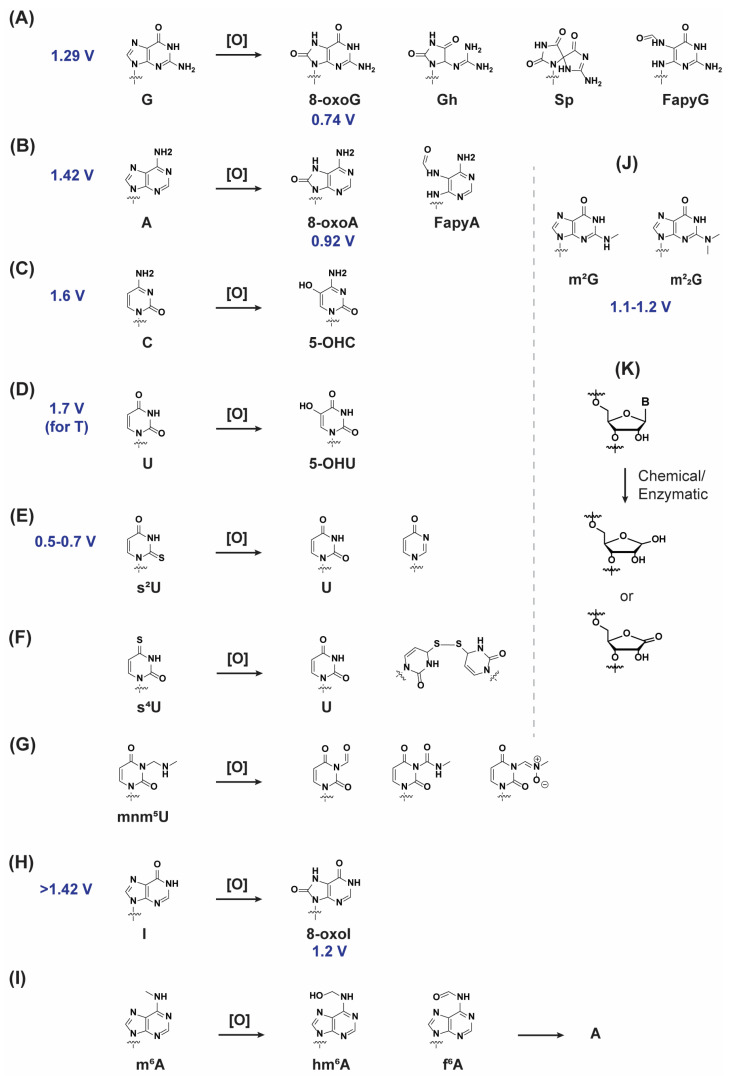
Structure of the nucleobases and their corresponding oxidation products. Oxidation potentials are coloured in blue and represented as Volts (V), reported versus Normal Hydrogen Electrode (NHE). Nucleobase code: (**A**) G = Guanine, 8-oxoG = 8-Oxoguanine, Gh = 5-guanidinohydantoin, Sp = spiroiminodihydantoin, FapyG = Formamidopyrimidineguanine; (**B**) A = Adenine, 8-oxoA = 8-Oxoadenine, FapyA = Formamidoadenine; (**C**) C = Cytosine, 5-OHC = 5-Hydroxycytosine; (**D**) U = Uracil, 5-OHU = 5-hydroxyuracil; (**E**) s^2^U = 2-Thiouracil; (**F**) s^4^U = 4-Thiouracil; (**G**) mnm^5^U = 5-Methylaminomethyluracil; (**H**) I = inosine, 8-oxoI = 8-Oxoinosine; (**I**) m^6^A = 6-Methyladenine, hm^6^A = 6-hydroxymethyladenine, f^6^A = 6-formyladenine; (**J**) m^2^G = 2-Methylguanine, m^2^_2_G = 2,2-dimethylguanine; (**K**) abasic site formation.

Examples of ROS include hydrogen peroxide (H_2_O_2_), hydroxyl radical (•OH), carbonate radical anion (CO_3_^•−^), superoxide (O_2_^•−^), singlet oxygen (^1^O_2_), ozone (O_3_), hypochlorous acid (HOCl), peroxyl radical (ROO•), and alkoxyl radical (RO•) [11]. Some of these species can be naturally handled by enzymes such as superoxide dismutases and reductases (2O_2_^•−^ + 2H^+^ + e^−^→ H_2_O_2_ or 2O_2_^•−^ + 2H^+^ → H_2_O_2_ + O_2_) [12], or glutathione peroxidases and catalase (2H_2_O_2_ → 2H_2_O + O_2_) [13,14], while other ROS, e.g., •OH [15,16], ^1^O_2_ [17], or CO_3_^•−^ [18,19], may not be captured by biological constituents and can react at diffusion controlled rates with biomolecules within their vicinity. The topic of other antioxidant enzymes and their relationship with various processes was recently reviewed [20]. Importantly, ROS are found in almost every organelle within the cell [21], and can also be generated from exogenous sources, e.g., exposure to ionizing or UV radiation [22]. A consequence of their reactivity is that ROS can induce changes in the structure of all nucleobases of DNA and RNA [23], with guanine (G) as a preferred target amongst the canonical nucleosides given its lower oxidation potential [24,25]. In fact, four oxidized nucleosides were first identified to be involved in oxidoreduction processes in yeast RNA, specifically 8-oxo-7,8-dihydropurines 8-oxoG and 8-oxoA, and 5-hydroxypyrimidines 5-OHU and 5-OHC (Scheme 1A–D) [26,27]. The oxidation of guanosine can lead to a variety of oxidatively generated modifications, and 8-oxo-7,8-dihydroguanosine (8-oxoG, or its 2′-deoxy homologue—Scheme 1A) is generally used as a biomarker, due to its established presence in DNA and RNA [28], and can be a modulator of biosystems broadly [29]. Structurally, oxidation at the C8 position changes the H-bonding pattern of the nucleobase, which can affect its base pairing ability [30], thus potentially impacting RNA–protein interactions.

Although other canonical nucleobases are less prone to oxidatively induced modification, these can also undergo the corresponding reactions, recently reviewed in the context of DNA [31], to give a range of different oxidative modifications at the nucleobase. It is reasonable to expect similar reactivity within strands of RNA. The oxidation of adenosine can also occur, where the reactivity of the purine ring is similar to that of guanosine and results in the formation of 8-oxo-7,8-dihydroadenosine (8-oxoA—Scheme 1B) as one of the main products (others can also be formed, such as Fapy•A, [32]). This has prompted various studies that focused on the development of antibodies [33], computational methodology to identify or evolve RNA–protein interactions [34,35], altered H-bonding interactions between helicases and oxidized nucleotides [36], as well as its impact on the structure of duplex regions within RNA [37]. It has also been incorporated and studied on a model of rRNA [38], and was found in patients with late-stage Alzheimer’s disease [39]. Its presence and repair on DNA have been thoroughly studied and are not discussed herein. Importantly, some of the reported reactivity, such as the formation of interstrand cross-links in DNA [40], may be potentially useful to detect or explore its role within RNA. The oxidation of pyrimidine rings (Scheme 1C,D) has been reported in the context of DNA, and their biochemical/biophysical properties have not been explored within RNA, with the exception of some experiments carried out on models of mRNA [41]. In addition, the natural occurrence of 5-hydroxylated pyrimidines has been established on rRNA [38,42], as well as their biogenesis [43]. This review does not focus on naturally occurring oxidative processes such as those observed in the transformation from 5-methylcytosine to 5-hydroxymethylcytosine (m^5^C → hm^5^C—[44,45]) or analogous processes involved in editing [46].

It is also worth mentioning that there are over 140 naturally occurring RNA modifications, and not much is known about their oxidation [47]. Examples include those of 2-methylguanosine (m^2^G) and 2,2-dimethylguanosine (m^2^_2_G, Scheme 1J), which have been shown to have a lower oxidation potential than G [48], with the former characterized in tRNA [49]. 2-Thiouridine (Scheme 1E,F) displays a lower oxidation potential than its canonical analogue and can also be oxidized by cytochrome c [50,51]. A related derivative, 5-methylaminomethyl-2-thiouridine (mnm^5^s^2^U, Scheme 1G), is more vulnerable to oxidative reactivity [52], while 5-methoxycarbonylmethyl-2-thiouridine (mcm^5^s^2^U) in eukaryotic cells is also prone to oxidation [53]. Similarly, 4-thiouridine (s^4^U) can undergo oxidation in vitro, which has been characterized within isolates of tRNA [54,55], with the oxidation of mnm^5^U also observed (Scheme 1G) [56]. Generally, thio-containing nucleosides possess distinct chemical and photophysical properties [57,58]. On the other hand, the oxidation of inosine is not likely to be a factor of biological relevance, given its higher oxidation potential (Scheme 1H) [59]. Oxidation reactions have been shown to have distinct reactivity for pseudouridine (Ψ) with potential for sequencing technologies [60], suggesting that their oxidation is possible and can lead to interesting biological properties within functional RNA, or alter rRNA modification profiles [61]. Another modification that can undergo oxidation is 6-methyladenosine (m^6^A), enzymatically or by reaction with ROS, which can result in its conversion to adenosine (Scheme 1I) [62,63].

Thus, the reactivity of different RNA modifications with ROS can potentially lead to other oxidatively modified nucleobases with distinct reactivity and biological implications. One example is the impact that nucleobase modification has on the stability of the glycosidic bond, where both oxidation and methylation can decrease bond strength [64,65,66] and have an impact on important processes such as the formation of abasic sites (Scheme 1K) [67]. In this regard, it is important to note that the chemical stability of RNA containing abasic sites is higher than that observed on DNA [68], and that such nucleobase-free modifications can occur as a consequence of oxidative modification on RNA [69]. These have also been observed in RNA oxidation catalyzed by cytochrome-c [70].

## 3. RNA Oxidation and 8-OxoG

Different types of RNA have been shown to be oxidized in vivo [71], including coding and non-coding RNAs, short (<200-nt) and long (>200-nt). Some examples include those involving microRNA (miRNA—[72,73,74,75]), ribosomal RNA (rRNA—[38,76]) that can lead to strand breaks [77], messenger RNA (mRNA—[41,78,79,80]), mRNA in plants [81], or mitochondrial RNA (mtRNA—[82]), as well as those in processes involved with viral RNA (vRNA—[83,84]). Most of the published studies use 8-oxoG as a biomarker (see Supplementary Materials, Section S2 for more about the detection and quantification of this oxidatively generated modification). In fact, the importance of oxidatively modified RNA and its presence in many instances has prompted the development of methods to effectively sequence for oxidatively modified nucleosides within RNA [48,85,86]. The detection and quantification of ROS and their products in biological systems is challenging, and has prompted the development of guidelines for their accurate characterization in such experiments [87]. In addition, recent reviews have addressed different aspects regarding RNA damage [88].

### 3.1. Handling of Oxidized RNA

As a consequence of structural differences (induced by reactions between ROS and nucleobases) and the biological implications arising from the presence of oxidatively generated lesions within RNA, it is reasonable to expect cellular processes to be in place to cope with such species. For example, folding mechanisms that reduce the amount of available guanine exposure to solvent, associated with increased susceptibility to react with ROS, have been identified [89]. The compartmentalization of oxidized RNA has also been shown as a potential coping mechanism, as well as for other type of RNA damage, perhaps for their eventual degradation [90,91]. The recruitment of oxidized RNA to stress granules along with their corresponding sorting and processing have also been proposed as a mechanism of action, with the direct involvement of various proteins, e.g., adenosine deaminase action on RNA (ADAR1), Staufen double-stranded RNA protein (STAU1), and Tudor staphylococcal nuclease (Tudor-SN) [92]. In addition, biochemical pathways to evade the incorporation of oxidized nucleotides (present in the nucleotide pool), through their hydrolysis to the corresponding nucleosides have been identified. These involve an *Arabidopsis* Nudix hydrolase (AtNUDX1) in plants [93], or guanylate kinases MutT and NUDT5 in bacterial and mammalian systems [94,95]. The importance of the efficiency of these processes is highlighted by the fact that some RNA polymerases can effectively incorporate 8-oxoGTP and exhibit distinct reactivity with 8-oxoATP [96]. On the other hand, inducing an excess of 8-oxoG nucleotides has been shown to lead to β-cell dysfunction [97], so it is plausible that their enzymatic hydrolysis is impacted and adverse reactions/processes to the cellular health are triggered. For the remainder of this review, we place focus on the ribonucleases that are known to be involved in the processing of oxidized RNA, and propose pathways that could be important in the handling of oxidatively modified RNA. The word oxidative ‘damage’ (a common term in DNA) is not used herein, as the possibility that oxidized RNA may function to trigger signalling pathways is plausible and potentially important. A review about RNA surveillance has been published and discusses some relevant RNA–protein interactions [98].

RNA quality control mechanisms are in place to target aberrant RNAs for their degradation [99], a function that is carried out by specific ribonucleases which can cleave strands of RNA in a processive or distributive manner [100] via cleavage of the phosphodiester bond within its backbone. Ribonucleases are also involved in RNA processing, including its degradation, and mRNA or noncoding-RNA turnover [101]. A distinction between RNA processing and RNA degradation can be inferred from their formed stable structures, bound proteins, and/or presence of base/sugar modifications. Of note are mechanisms that have evolved to deal with defective mRNAs, such as nonsense-mediated decay (NMD), which refers to the process of targeting aberrant mRNAs for decapping and degradation, or no-go decay (NGD), which refers to endonucleolytic cleavage of mRNA with strong pauses in elongation [102], and staufen mediated decay (SMD) which targets double-stranded RNA [103]. In this regard, the oxidation of NAT8L mRNA has been shown to reduce protein synthesis [79], displaying >1000 fold decreased peptide bond formation when 8-oxoG is present within mRNA [41,104]. In addition, mRNA containing 8-oxoG has also been shown to affect translational fidelity [78]. For a review about the impact of other modifications on mRNA see [105]. At this point, it is also important to note that the functions of ribonucleases go well beyond those of solely RNA degradation and with a rich history in the community [106,107]; however, those are not discussed in this review. For examples about ribonucleases involved in other processes see Supplementary Materials, Section S3 and Table S1.

### 3.2. Interactions Between Oxidized RNA and Ribonucleases or RNA-Binding Proteins

With regard to ribonuclease involvement in the handling of oxidatively modified RNA, turnover of oxidized RNA has been shown in epithelial cells, where a portion of 8-oxoG in RNA is cleared following the removal of oxidative stress, providing evidence of RNA degradation [108], as also observed on HeLa cells [109]. Furthermore, the formation of 8-oxoG has been shown to coincide with a decrease in rRNA [110,111]. An association between oxidative stress and tRNA cleavage has also been observed [112]. Simms et al. discussed chemical modifications, some of which arise from oxidative stress and touch upon mRNA, tRNA, and rRNA oxidation, as well as cellular handling of damaged RNA [113,114]. As delineated within Table 1 (top), the three ribonucleases that have been reported to be involved in the enzymatic cleavage of oxidized RNA are Xrn-1, ISG20, and PNPase. It is important to note that PNPase is part of a larger body composed of proteins with various functions, namely the degradosome, although it can also act independently [115] or in association with other binding proteins such as Hfq and poly(A) polymerase I [116], as well as helicase RhIB [117]. In addition the exosome is another multi-subunit complex essential for RNA processing [118,119]; and other ribonucleases with various roles, including activity under stress, are mentioned in Table 1 (bottom). 

Some of the handling of oxidized mRNA has been shown to involve the ribosome, displaying decreased peptydil transfer rates [104]. In this case, Xrn-1 was shown to be responsible for its degradation, and subject to NGD in a coordinated mechanism with Dom34, which is known to expedite RNA degradation [132,133] by binding to the A-site of the ribosome, and recruiting the exosome. In fact, Dom34 can form a surveillance complex with RNase L and act in the recognition of exogenous mRNA for its subsequent degradation, as a potential antiviral defence mechanism [134]. The combination of Xrn-1 and Dom-34 seems to be important as Xrn-1 has been shown to stall upon encountering 8-oxoG within strands of RNA, in the absence of Dom34 [135]; however, more work is needed to establish the exact interactions between oxidized RNA and these proteins. Importantly, exoribonuclease family Xrn1/Xrn2 carries out enzymatic hydrolysis of RNA within the nucleus [136]. Another protein of interest is the AU-rich element/poly(U)-binding/degradation factor AUF1 (also called HNRNPD, since it is a heterogeneous nuclear ribonucleoprotein) which has been shown to participate in the decay of oxidized mRNA [137,138]; however, the specific ribonuclease has not been determined. In this regard, AUF1 is an RNA binding protein known to be involved in RNA folding [139] and with functions associated with mRNA turnover. Other proteins that bind with lower affinity to oxidized RNA include HNRNPC and PNP. Amongst these, polynucleotide phosphorylase (PNPase and its human homologue PNPT1) is of particular interest, as it has been shown to display preferential binding to oxidized RNA, specifically RNA containing 8-oxoG [140,141]. This was reviewed recently [142]; the S1 domain plays an important role in the binding and handling of RNA containing 8-oxoG in the cytoplasm and mitochondria [143]. PNPase and DEAD box RNA helicase (DR_0335/Rh1B) are involved in targeting RNAs containing 8-oxoG and grant resistance to oxidative stress in *Deinococcus radiodurans* [144]. Interestingly, as previously described with Xrn-1, PNPase (expressed from *E. coli*) has also been shown to stall upon encountering 8-oxoG within strands of RNA. In this case, Cryo-EM structures displayed interactions between residues in the active site of PNPase and 8-oxoG that are responsible for the stalling behaviour [145]. Therefore, other factors might be involved that allow for the processing of the oxidized substrates. For example, the role of the three main bacterial ribonucleases (RNase II, RNase R, PNPase) has been studied, with the conclusion that they have different effects on cellular motility and different RNA targets [146]. It is also important to note that PNPase is a subunit of the degradosome, a multiprotein complex, and is necessary for oxidative stress under certain conditions [147]. In addition, the degradosome also contains other ribonucleases such as RNase E, an endonuclease that complements the exonuclease activity of PNPase [148], although this relationship is yet to be explored in the context of handling oxidized RNA. Contrary to the observed stalling using bacterial PNPase (*E. coli*), human mitochondrial PNPase was recently shown to have evolved with distinct structural features that allow for efficient degradation of RNA containing 8-oxoG [149]. This brings into question the role of PNPase within different organisms and possibly distinct mechanisms that handle oxidized RNA.

Another ribonuclease that was found to be involved in the degradation of oxidized RNA is ISG20 (also known as HEM45) [124,125]. A link between the pathophysiology of acute kidney injury and oxidation of RNA was established by measuring the upregulation of ISG20 in kidneys of AKI model mice. Furthermore, levels of oxidized RNA were reduced on models overexpressing ISG20, with the conclusion that ISG20 takes an active role in the degradation of oxidatively modified RNA. While this exonuclease is commonly associated with an antiviral response [150], recent reports have studied orthologs with a role in ribosomal biogenesis [151], which suggests that the activity of this exonuclease is broader than our current knowledge.

Other than Xrn-1, PNPase, and ISG20, there are no reports that have specifically tested for the degradation of oxidized RNA, which brings into question the potential role of other protein complexes, such as the exosome, to perform these processing functions. For example, the nuclear-specific subunit Rrp4 (also called Dis3), found within the exosome, has the capability to direct up to three RNA substrates for degradation [152]. On this note, it is worth noting that the exosomes from different organisms can vary in structure and function [153], and thus distinct mechanisms could be at play. Another important factor is the role of RNA-binding proteins (RBPs) that can form complexes with oxidized RNA, a topic that was recently reviewed [154] and is briefly summarized within the Supporting Materials (Section S4).

The role of helicases is also worth considering since these are in part responsible for facilitating RNA degradation, amongst other functions, and are present within the RNA degradation complexes [155,156,157,158]. There are no available reports that test the impact of oxidative stress (or presence of oxidatively generated modifications) in the context with helicases; however, at least one helicase has been found to be associated with RNA containing 8-oxoG [143], although other experiments displayed independence between PNPase degradation of oxidized RNA and the activity of helicase RhIB [141]. In addition, the presence of modifications that alter duplex stability is known to affect helicase activity [159], which has prompted a view of their role as protective agents against oxidative stress [160]. Another aspect of potential relevance is the relationship between miRNA oxidation and proteins associated with miR biogenesis [161], as well as with siRNA [162]. Amongst the studies that have reported on oxidized miRNA ([72,73,74,75,163] all containing 8-oxoG), the stage at which miRNA is oxidized has not been established (nuclear pri-miRNA, cytoplasmic pre-miRNA or mature miRNA). This brings into question the potential for altered RNA–protein interactions in miRNA biogenesis involving processing (DROSHA, DGCR8, spliceosome and AGO2, DICER) and/or export (Exportin 1 or 5, RanGTP) [164], following RNA oxidation.

It is also important to note that the landscape of RNA–enzyme interactions is known to vary significantly in the presence/absence of oxidative stress [165], which indicates that distinct mechanisms are in place to handle oxidized RNA. This includes the potential role of extracellular vesicles to clear the cells of oxidized RNA, with a known role with other types of RNA [166], although this is yet to be studied. In this regard, extracellular ribonucleases such as those in the RNase A and T families could play an important role; these are known to hydrolyze/process stress-induced tRNAs, specifically from oxidative stress [167]. Therefore, while the reactivity between these RNases and RNA containing 8-oxoG has been shown to vary (RNase T_1_ does not cleave at 8-oxoG sites, while RNase A does, in some sequence contexts [168]), it is possible that the degradation of extracellular RNAs by these ribonucleases is effective. In addition to degradation, apoptosis [169] and autophagy [170] may play a role in the clearance of cells or cellular materials that accumulate oxidized RNA products.

**Figure 1:**
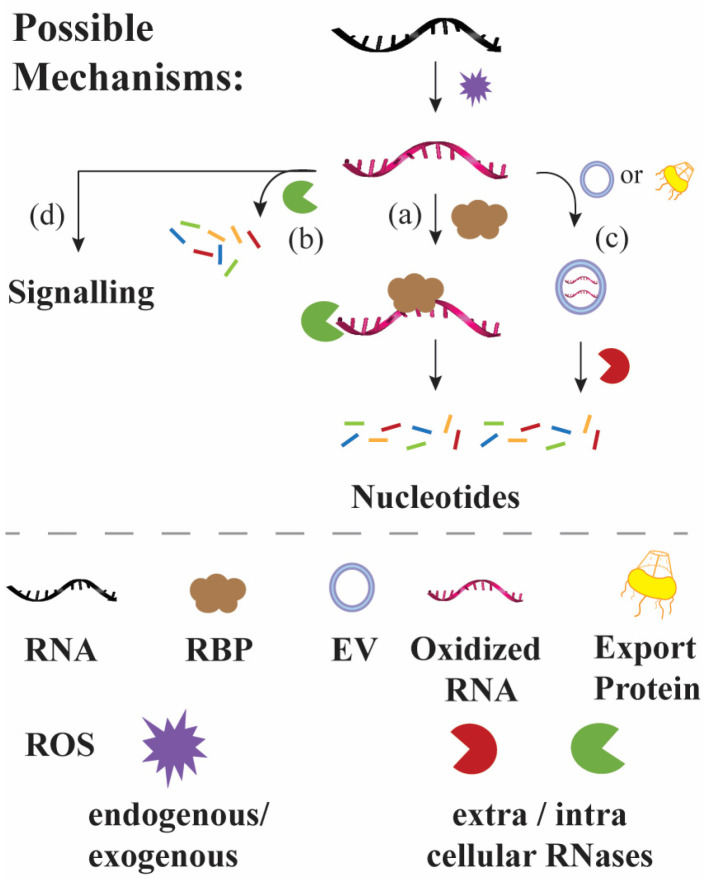
Simplified picture illustrating known and potential intra- and extracellular paths in charge of handling oxidized RNA (left) and mechanisms mentioned in this review that led to the degradation of oxidized RNA, some which have been reported ((a), (b), (d)) and others that were hypothesized herein (c). (a) Specific recognition of oxidized RNA by binding proteins; (b) enzymatic degradation of oxidized RNA by ribonucleases; (c) intra-to-extracellular export; and (d) signalling mechanisms.

Another point of consideration is the potential repair of oxidized RNA, where enzymes involved in DNA repair have been shown to be involved in RNA processing; this relationship and other data suggesting a possible repair have been recently reviewed [171]. Our group has found that ribonucleases exhibit different reactivity towards the enzymatic cleavage of oxidized RNA [135,145,168], and it is worth noting that other groups have also used altered reactivity of RNA containing 8-oxoG with other enzymes [75], as well as with naturally occurring modifications; e.g., pseudouridine can also alter the reactivity of nucleases towards RNA containing this modification [172]. Thus, a full picture that describes the handling of oxidized RA within different cellular environments is yet to be fully elucidated. As depicted in Figure 1, mechanisms that can be involved in the handling of oxidized RNA include (a) specific recognition of oxidized RNA followed by degradation, (b) direct enzymatic hydrolysis, (c) export to extracellular environment and subsequent degradation, (d) repair or (e) triggering of a cellular signal.

## 4. Conclusions

RNA is a highly diverse biopolymer that is involved in a plethora of important biological functions, which can be orchestrated as a function of their sequence. Therefore, alteration of the nucleobase(s) by oxidative modification may lead to dysfunction, and/or trigger a signal that results in the handling of the oxidized RNA, or lead to apoptosis, among other outcomes. One of the most common intracellular mechanisms involves the enzymatic hydrolysis of the oxidatively modified RNA by ribonucleases, which include Xrn-1, ISG-20, and PNPase. How these oxidized RNAs are recognized remains an area to be further studied; however, there are a number of studies that have found proteins with high affinity towards these species. More research and information are needed to create a clear picture of how oxidized RNA is handled, as well as its biological implications.

## Figures and Tables

**Table 1:** Ribonucleases reported to be involved in the hydrolysis/phosphorolysis/degradation of oxidized RNA (containing 8-oxoG) (top), and ribonucleases within the processing bodies that may also handle these species (bottom).

Ribonuclease	Localization	Mechanism ^§^	Target *	Refs. ^‡^
Xrn-1, Xrn-2	Nucleus, cytoplasm	Processive exo 5′→3′ ss-RNA	mRNA, vRNA, tRNA, siRNA	[120,121]
PNPase	Mitochondria, cytoplasm	Processive exo 3′→5′ ss-RNA	Bulk RNA, mRNA, rRNA, small RNA	[122,123]
ISG20	Cytosol, nucleus	Processive exo 3′→5′ ss-RNA	vRNA, rRNA	[124,125]
Other ribonucleases with important RNA degradation roles.				
RNase E (within degradosome)	Inner membrane	endo	mRNA, tRNA, rRNA, sRNA	[126,127,128]
RNase R (RNase II homologue)	Cytoplasm	3′→5′ exo rRNA, tRNA, mtRNA, mRNA	All types	[129,130,131]

* These represent some of the RNA targets; other types of RNA may also function as substrates. ^‡^ The references are complementary to those found throughout the text and focus on function and localization; these do not necessarily associate with damaged/oxidized RNAs. ^§^ exo/endo stands for exonucleolytic/endonucleolytic activity.

## Data Availability

No new data were created or analyzed in this study.

## Supplementary Materials

The following supporting information can be downloaded at: https://www.mdpi.com/article/10.3390/biom16040564/s1, Supporting Materials Sections S1–S4 and Table S1. Proteins known to bind to and/or react with oxidized RNA, where provided references focus on the RNA binding domain corresponding to each protein.

## Abbreviations

The following abbreviations are used in this manuscript (alphabetical order):

m_2_G	2-Methylguanosine
m_2_^2^G	2,2-Dimethylguanosine
S4U	4-Thiouridine
hm^5^C	5-Hydroxymethylcytosine
mcm^5^s^2^U	5-Methoxycarbonylmethyl-2-thiouridine
mnm^5^s^2^U	5-Methylaminomethyl-2-thiouridine
m^5^C	5-Methylcytosine
m^6^A	6-Methyladenosine
8-oxoA	8-Oxo-7,8-dihydroadenosine
8-oxoATP	8-Oxo-7,8-dihydroadenosine triphosphate
8-oxoG	8-Oxo-7,8-dihydroguanosine
8-oxoGTP	8-Oxo-7,8-dihydroguanosine triphosphate
ADAR1	Adenosine deaminase action on RNA
RO•	Alkoxyl radical
APE1	Apurinic/apyrimidinic endonuclease 1
AtNUDX1	Arabidopsis Nudix hydrolase
AUF1	AU-rich element/poly(U)-binding/degradation factor
CTD	C-terminal domain
CO_3_^•−^	Carbonate radical anion
CSD	Cold-shock domain
DR_0335/Rh1B	DEAD box RNA helicase
DEDD	Death Effector Domain containing DNA binding protein
dsRNA	Double stranded RNA
DOM34	Duplicate of multilocus
Xrn	Exoribonuclease
exRNA	Extracellular RNA
gRNA	Guide RNA
HNRNP	Heterogeneous nuclear ribonucleoprotein
H_2_O_2_	Hydrogen peroxide
•OH	Hydroxyl radical
HOCl	Hypochlorous acid
ISG20	Interferon-stimulated gene 20
IGF2BP	Insulin-line growth factor 2 mRNA binding protein
KH	Kinase homology
mRNA	Messenger RNA
miRNA	microRNA
mtRNA	Mitochondrial RNA
NTD	N-terminal domain
NAT8L	N-acetyl aspartate transferase 8 like
NBS	Nijmegen breakage syndrome
NGD	No-go decay
NMD	Nonsense mediated decay
NUDT5	Nudix Hydrolase 5
O_3_	Ozone
ROO•	Peroxyl radical
PCB or PCBP	Poly(rC)-binding protein
PNPase	Polyribonucleotide Phosphorylase
PNP	Purine Nucleoside Phosphorylase
PNPT1	Polyribonucleotide nucleotidyltransferase 1
ROS	Reactive oxygen species
RNase	Ribonuclease
RNP	Ribonucleoprotein
rRNA	Ribosomal RNA
Rrp	Ribosomal RNA-processing protein
RBPs	RNA-binding proteins
RhlB	RNA Helicase B
RRM	RNA recognition motif
ssRNA	Single Stranded RNA
^1^O_2_	Singlet oxygen
SMD	Staufen mediated decay
sRNA	Small RNA
siRNA	Small interfering RNA
STAU1	Staufen double-stranded RNA protein
O_2_^•−^	Superoxide
tRNA	Transfer RNA
Tudor-SN	Tudor staphylococcal nuclease
tiRNA	tRNA-derived stress-induced small RNA
vRNA	Viral RNA
YB-1	Y-box binding protein
