# Supplementary material for: Ribonucleobase Oxidation and Ribonucleases Involved in the Degradation of Oxidized RNA
Source: Biomolecules. 2026 Apr 10;16(4):564. doi: 10.3390/biom16040564 (PMC13113920; doi:10.3390/biom16040564)
Supplement: Supplementary file 1 [file biomolecules-16-00564-s001.zip › biomolecules-4157626-supplementary.pdf]

Supporting Information

Review

Ribonucleobase Oxidation and Ribonucleases Involved in the Degradation of Oxidized RNA

Dagoberto Grijalva-Flores [1] and Marino J. E. Resendiz [1,*]

[1] Department of Chemistry, University of Colorado Denver, Science Building 1151 Arapahoe St, Denver, CO 80204, USA
* Correspondence: marino.resendiz@ucdenver.edu; Tel.: (303-315-7658, MJER)

Index

Page **Content**

Section S1……………………………..Paragraph/references about oxidation of RNA and disease as
well as other biological processes
Section S2……………………………..Paragraph/references about detection and quantification of
8-oxoG
Section S3……………………………..Paragraph/references about ribonucleases involved in other
processes
Section S4…………………………..Paragraph and table about proteins binding to oxidized RNA
7-10..………………………..References

Section S1. Oxidation of RNA and disease as well as other biological processes

Processes affected/associated with/by this include genome stability, [1] aging, [2,3] and life-style (for example substance abuse [4]), as well as diseases such as diabetes, [5,6] cardiac related conditions, [7-9] neurodegenerative diseases, [10-13] psychiatric disorders,[14] bipolar disorder,[15,16] multiple sclerosis,[17] and cancer development, [18] amongst others. In addition, cigarette smoke,[19] the consumption of certain antibiotics, [20] exposure to pollutants or environmental factors,[21] or specialized treatments,[22] can also induce the generation of ROS and lead to the corresponding oxidation of RNA. In addition, different studies have placed emphasis on this topic, with varying results, for example showing that exercise might be a factor that contributes in avoiding oxidation of RNA.[23]

Section S2. **Detection and quantification of 8-oxoG.**

Detection of oxidatively generated modifications varies from using qualitative methods such as quantification of oxidized nucleosides from processed RNA, to sequencing that involves erroneous incorporation of nucleotides using reverse transcription, since 8-oxoG can form stable base pair interactions with C and A.[24] Quantities of 8-oxoG and 8-oxodG can be measured in urine and plasma.[25] Its impact when 8-oxoG is present in mRNA adversely impacts translation.[26] In another study 8-oxoG was quantified in tRNA as a consequence of environmental factors (acetamiprid exposure, [27]). In plants, oxidative stress induced by Cadmium can also lead to the oxidation of RNA, as measured through 8-oxoG content.[28]

Section S3. Ribonucleases involved in other processes

In addition, ribonucleases are also important in t-RNA [29], rRNA [30] guide RNA (gRNA, [31]) and miRNA [32, 33] biogenesis and function, to mention a few types of RNA of varying sizes, structure, and cell localization. Viral RNAs can also be degraded by some of these ribonucleases, e.g., Xrn-1, RNase L, and cofactors of the exosome [34] by degrading, or repressing the function of dsRNAs.

Section S4. Protein binding to oxidized RNA

Ribonucleases can bind to RNA through different domains, and although there is knowledge about some of the most common RBP domains, a better understanding and classification of other RBP domains is still needed,[35] which could shed light on how these proteins may associate with oxidized RNA. A list of proteins reported to bind to oxidized RNA is included in TableS1.

**Ribonuclease**	**Target***	**Ref.**
Degrade oxidized RNAs		
Polynucleotide Phosphorylase (PNPase)	sRNA, lncRNA (all Types of RNA)	[36] [37]
Xrn-1	mRNA	[38]
ISG20	vRNA	[39]
Bind to oxidized RNA		
YB-1	Pre-mRNA	[40,41]
IGF2BP	mRNA, ssRNA, dsRNA, (SL)RNA	[42, 43]
hnRNPD	mRNA	[44, 45]
PCBP1	mRNA	[46-48]
PCBP2	mRNA	[48,49]
APE 1	miRNA, rRNA	[50-53]

Table S1. Proteins known to bind and/or react with oxidized RNA, where provided references focus on the RNA binding domain corresponding to each protein.

* This includes some of the common RNA targets, however some of these proteins have many functions and could interact with other types of RNA.

Hayakawa et al., also discovered that the Y box-binding protein 1 (YB-1) can preferentially bind to RNA containing 8-oxoG.[40] While its role with handling of oxidized RNA is unknown, this protein is associated with the DNA repair machinery,[54] with higher binding affinity towards RNA over DNA via its cold-shock domain (CSD),[55] a function that has made it a druggable candidate due to its involvement in apoptosis and RNA degradation.[56] Thus, it is likely that indeed, YB-1 plays an important role in the handling of oxidized RNA and will be interesting to further study if it can recognize these entities in a selective manner. In looking at association of proteins with oxidized RNA, Apurinic/apyrimidinic endonuclease 1 (APE1, associated with the DNA repair machinery) influences RNA quality control, specifically RNA containing 8-oxoG.[50] Another approach that used a crosslinking strategy revealed that RNA containing 8-oxoG associates with insulin-like growth factor-2 mRNA-binding proteins (IGF2BP) family proteins, [42] which typically recognize mRNA modified by methylation; as well as heterogeneous nuclear ribonucleoprotein D (hnRNPD), proposed to be a quality control protein [44]. Furthermore, PCBP1 and PCBP2, members of the poly(C)-binding protein family have been shown to bind RNA containing 8-oxoG lesions, [57] and their role in suppressing or increasing apoptosis under oxidative stress was reported. On this note, it is interesting that these two proteins are directly involved in response to managing intracellular iron flux, [43] since iron is a key component of the Fenton reaction which results in the formation of ROS and could have a connection to its binding of oxidized RNA.

References

1. Olatunji, M.; Liu, Y. RNA damage and its implications in genome stability. *DNA Repair*, **2025**, *147*, 103821.
2. Hemagirri, M.; Sasidharan, S. Biology of aging: Oxidative stress and RNA oxidation. Mol. Biol. Rep. 2022, 49, 5089-5105.
3. Stirpe, M.; Palermo, V.; Ferrari, M.; Mroczek, S.; Kufel, J.; Falcone, C.; Mazzoni, C. Increased levels of RNA oxidation enhance the reversion frequency inn aging pro-apoptotic yeast mutants. *Apoptosis*, **2017**, *22*, 200-206.
4. Viola, T. W.; Orso, R.; Florian, L. F.; Garcia, M. G.; Gomes, M. G. S.; Mardini, E. M.; Niederauer, J. P. O.; Zaparte, A.; Grassi-Oliveira, R. Effects of substance use disorder on oxidative and antioxidative stress markers: A systematic review and meta-analysis. *Addict. Biol*. **2023**, *28*, e13254.
5. Cejvanovic, V.; Kjær, L. K.; Bergholdt, H. K. M.; Kenriksen, T.; Weimann, A.; Ellervik, C.; Poulsen, H. E. RNA oxidation and iron levels in patients with diabetes. *Free Rad. Biol. Med*. **2018**, *129*, 532-536.
6. Broedbaek, K.; Siersma, V.; Henriksen, T.; Weimann, A.; Petersen, M.; Andersen, J. T.; Jimenez-Solem, E.; Hansen, L. J.; Henriksen, J. E.; Bonnema, S. J.; Olivarius, N. F.; Friis, S.; Poulsen, H. E. Urinary markers of nucleic acid oxidation and cancer in type 2 diabetes. *Redox Biol*. **2015**, *4*, 34-39.
7. Liang, Y-D.; Liu, Q.; Du, M-H.; Liu, Z.; Yao, S-M.; Zheng, P-P.; Wan, Y-H.; Sun, N.; Li, Y-Y.; Liu, J-P.; Luo, Y.; Cai, J-P.; Yang, J-F.; Wang, H. Urinary 8-oxo-7,8-dihydroguanosine as a potential of frailty for elderly patients with cardiovascular disease. *Free Rad. Biol. Med.* **2020**, *152*, 248-254.
8. Liu, T.; Cai, J-P.; Zhang, L-Q.; Sun, N.; Cui, J.; Wang, H.; Yang, J-F. The mechanisms of RNA oxidation involved in the development of heart failure. *Free Rad. Res.* **2019**, *53*, 910-921.
9. Li, Y.; Wang, X. The role of DNA and RNA guanosine oxidation in cardiovascular diseases. *Pharmacol. Res*. **2024**, *204*, 107187.
10. Wheeler, H. B.; Madrigal, A. A.; Chaim, I. A. Mapping the future of oxidative RNA damage in neurodegeneration: Rethinking the status quo with new tools. *Proc. Natl. Acad. Sci. U. S. A.* **2024**, *121*, e2317860121.
11. Rodriguez,Callejas, J. D.; Cuervo-Zanatta, D.; Rosas-Arellano, A.; Fonta, C.; Fuchs, E.; Perez-Cruz, C. Loss of ferritin-positive microglia relates to increased iron, RNA oxidation, and dystrophic microglia in the brains of aged male marmosets. *Am. J. Primatol*. **2019**, *81*, e22956.
12. Weidner, A. M.; Bradley, M. A.; Beckett, T. L.; Niedowicz, D. M.; Dowling, A. L. S.; Matveev, S. V.; LeVine, H.; Lovell, M. A.; Murphy, M. P. RNA oxidation adducts 8-OHG and 8-OHA change with Aβ42 levels in late-stage Alzheimer's disease. *PLoS ONE*, **2011**, *6*, e24930.
13. Nunomura, A.; Lee, H-g.; Zhu, X.; Perry, G. Consequences of RNA oxidation on protein synthesis rate and fidelity: implications for the pathophysiology of neuropsyciatric disorders. *Biochem. Soc. Trans.* **2017**, *45*, 1053-1066.
14. Maluach, A. M.; Misquitta, K. A.; Prevot, T. D.; Fee, C.; Sibille, E.; Banasr, M.; Andreazza, A. C. Increased neuronal DNA/RNA oxidation in the frontal cortex of mice subjected to unpredictable chronic mild stress. *Chronic Stress*, **2017**, *1*, 2470547017724744.
15. Jacoby, A. S.; Vinberg, M.; Poulsen, H. E.; Kessing, L. V.; Munkholm, K. Increased DNA and RNA damage by oxidation in patients with bipolar I disorder. *Transl. Psychiatry*, **2016**, *6*, e867.
16. Knorr, U.; Simonsen, A. H.; Roos, P.; Weimann, A.; Henriksen, T.; Christensen, E-M.; Vinberg, M.; Mikkelsen, R. L.; Kirkegaard, T.; Jensen, R. N.; Akhøj, M.; Forman, J.; Poulsen, H. E.; Hasselbach, S. G.; Kessing, L. V. Cerebrospinal fluid oxidative stress metabolites in patients with bipolar disorder and healthy controls: a longitudinal case-control study. *Transl. Psy*. **2019**, *9*, 325.
17. Kharel, P.; McDonough, J.; Basu, S. Evidence of extensive RNA oxidation in normal appearing cortex of multiple sclerosis brain. *Neurochem. Int.* **2016**, *92*, 43-48.

18. Gao, X.; Holleczek, B.; Cuk, K.; Zhang, Y.; Anusruti, A.; Xuan, Y.; Xu, Y.; Brenner, H.; Schöttker, B. Investigation on potential associations of oxidatively generated DNA/RNA damage with lung, colorectal, breast, prostate and total cancer incidence. *Sci. Rep.* **2019**, *9*, 7109.
19. Deslee, G.; Adair-Kirk, T. L.; Betsuyaku, T.; Woods, J. C.; Moore, C. H.; Gierada, D. S.; Conradi, S. H.; Atkinson, J. J.; Toennies, H. M.; Battaile, J. T.; Kobayashi, D. K.; Patterson, G. A.; Holtzman, M. J.; Pierce, R. A. Cigarette smoke induces nucleic –acid oxidation in lung fibroblasts. *Am. J. Respir. Cell. Mol. Biol.* **2010**, *43*, 576-584.
20. Larsen, E. L.; Cejvanovic, V.; Kjaer, L. K.; Pedersen, M. T.; Popik, S. D.; Hansen, L. K.; Andersen, J. T.; Jimenez-Solem, E.; Broedbaek, K.; Petersen, M.; Weimann, A.; Henriksen, T.; Lykkesfeldt, J.; Torp-Pedersen, C.; Poulsen, H. E. Claritrhomycin, trimethorprim, and penicillin and oxidative nucleic acid modifications in humans: randomized, controlled trials. *Br. J. Clin. Pharmacol.* **2017**, *83*, 1643-1653.
21. Gonzales-Rivera, J. C. Sherman, M. W.; Wang, D. S.; Chuvalo-Abraham, J. C. L.; Ruiz, L. H.; Contreras, L. M. RNA oxidation in chromatin modification and DNA-damage response following exposure to formaldehyde. *Sci. Rep.* **2020**, *10*, 16545.
22. Nielsen, B.; Cejvanovic, V.; Wörtwein, G.; Hansen, A. R.; Marstal, K. K.; Weimann, A.; Bjerring, P. N.; Dela, F.; Poulsen, H. E.; Jørgensen, M. B. Increased oxidation of RNA despite reduced mitochondrial respiration after chronic electroconvulsive stimulation of rat brain tissue. *Neurosci. Lett.* **2019**, *690*, 1-5.
23. Larsen, E. L.; Karstoft, K.; Poulsen, H. E. Exercise and RNA oxidation. In Oxidative sustress in exercise physiology, Chapter 8, 2022, pp 95-102, CRC Press, 1st Edition.
24. Poulsen, H. E.; Weimann, A.; Henriksen, A.; Kjaer, L. K.; Larsen, E. L.; Carlsson, E. R.; Christensen, C. K.; Brandslud, I.; Fenger, M. Oxidatively generated modifications to nucleic acids in vivo: Measurements in urine and plasma. *Free Rad. Biol. Med.* **2019**, *145*, 336-341.
25. Thomas, E. N.; Simms, C. L.; Keedy, H. E.; Zaher, H. S. Insights into the base-pairing preferences of 8-oxoguanosine on the ribosome. *Nucleic Acids Res.* **2019**, *47*, 9857-9870.
26. Zhang, H-X.; Yu, D.; Sun, J-F.; Zeng, L.; Wang, C-Y.; Bai, L-P.; Zhu, G-Y.; Jiang, Z-H.; Zhang, W. An integrated approach to evaluate acetamiprid-induced oxidative damage to tRNA in human cells based on oxidized nucleotide and tRNA profiling. *Environ. Int.* **2023**, *178*, 108038.
27. Chmielowska-Bak, J.; Izbiańska, K.; Ekner-Grzyb, A.; Bayar, M.; Deckert, J. Cadmium stress leads to rapid increase in RNA oxidative modifications in soybean seedlings. *Front. Plant Sci.* **2018**, *8*, 2219.
28. Cordes, J.; Zhao, S.; Engel, C. M.; Stingele, J. Cellular responses to RNA damage. *Cell*, **2025**, *188*, 885-900.
29. Elder, J. J. H.; Papadopoulos, R.; Hayne, C. K.; Stanley, R. E. The making and breaking of rRNAs by ribonucleases. *Trends Genet.* **2024**, *40*, 511-525.
30. Lan, P.; Zhou, B.; Tan, M.; Li, S.; Cao, M.; Wu, J.; Lei, M. Structural insight into precursor ribosomal RNA processing by ribonuclease MRP. *Science*, **2020**, *369*, 656-663.
31. Gao, Y.; Liu, H.; Zhang, C.; Su, S.; Chen, Y.; Chen, X.; Li, Y.; Shao, Z.; Zhang, Y.; Shao, Q.; Li, J.; Huang, Z.; Ma, J.; Gan, J. Structural basis for guide RNA trimming by RNase D ribonuclease in Tryponosoma brucei. *Nucleic Acids Res.* **2021**, *49*, 568-583.
32. Maurin, T.; Cazalla, D.; Yang, Jr-S.; Bortolamiol-Becet, D.; Lai, E. C. RNase III-independent microRNA biogenesis in mammalian cells. *RNA*, **2012**, *18*, 2166-2173.
33. Fernandez, N.; Cordiner, R. A.; Young, R. S.; Hug, N.; Macias, S.; Cáceres, J. F. Genetic variation and RNA structure regulate microRNA biogenesis. *Nature Commun*. **2017**, 8, 15114.
34. Watkins, J. M.; Burke, J. M. RNase L-induced bodies sequester subgenomic flavivirus RNAs to promote viral RNA decay. *Cell Rep.* **2024**, *43*, 114694.
35. Corley, M.; Burns, M. C.; Yeo, G. W. How RNA-binding proteins interact with RNA: Molecules and mechanisms. *Mol. Cell*, **2020**, *78*, 9-29.

36. Wong, A. G.; McBurney, K. L.; Thompson, K. J.; Sickney, L. M.; Mackie, G. A. S1 and KH domains of polynucleotide phosphorylase determine the efficiency of RNA binding and autoregulation. *J. Bacteriol*. **2013**, *195*, 2021-2031.
37. Bycroft, M.; Hubbard, T. J. P.; Proctor, M.; Freund, S. M. V.; Murzin, A. G. The solution structure of the S1 RNA binding domain: a member of an ancient nucleic acid-binding fold. *Cell*, **1997**, *88*, 235-242.
38. Jinek, M.; Coyle, S. M.; Doudna, J. A. Coupled 5′ nucleotide recognition and processivity in Xrn-1-mediated mRNA decay. *Mol. Cell*, **2011**, *41*, 600-608.
39. Horio, T.; Murai, M.; Inoue, T.; Hamasaki, T.; Tanaka, T.; Ohgi, T. Crystal structure of human ISG20, an interferon-induced antiviral ribonuclease. *FEBS Lett.* **2004**, *577*, 111-116.
40. Harakawa, H.; Uchiumi, T.; Fukuda, T.; Ashikuza, M.; Kohno, K.; Kuwano, M.; Sekiguchi, M. Binding capacity of huban YB-1 protein for RNA containing 8-oxoguanine. *Biochemisty*, **2002**, *41*, 12739-12744.
41. Yang, X.; Zhu, H.; Mu, S.; Wei, W.; Yuan, X.; Wang, M.; Liu, Y.; Hui, J.; Huang, Y.; Crystal structure of a Y-box binding protein 1 (YB-1)-RNA complex reveals key features and residues interacting with RNA Crystal structure of YB-1-RNA complex. *J. Biol. Chem.* **2019**, *294*, 10998-11010.
42. Villers, J.; Smith, MC, DeLiberto, A. N.; Arguello, E.; Nyaanga, J.; Kleiner, R. E. Chemoproteomic profiling of 8-oxoguanosine sensitive RNA-Protein interactions. *Biochemistry*, **2023**, *62*, 3411-3419.
43. Marianne K.S.; Ulshöfer, C.J.; Schneider, Tim.; Schlundt, A.; Structures and target RNA preferences of the RNA-binding protein family of IGF2BPs: An overview, *Structures*, **2021**, *29*, 787-803.
44. Hayakawa, H.; Fujikane, A.; Ito, R.; Matsumoto, M.; Nakayama, K. I.; Sekiguchi, M. Human proteins that specifically bind to 8-oxoguanin-containing RNA and their responses to oxidative stress. *Biochem. Biophys. Res. Commun*. **2010**, *402*, 220-224.
45. Nagata, T.; Kurihara, Y.; Matsuda, G.; Saeki, J.; Kohno, T.; Yanagida, Y.; Ishikawa, F.; Uesugi, S.; Katahira, M.; Structure and interactions with RNA of the N-terminal UUAG-specific RNA-binding domain of hnRNP D011, *J. Mol. Biol.* **1999**, *287*, 221-237.
46. Goda, G. A.; Forbes, K.; Sullivan, M. E.; Eramo, G. A. Breen C.; Porter, D. F.; Khavari, P. A.; Dominguez, D. Aleman, M. M. Iron-sensitive RNA regulation by poly C-binding proteins. *Nucleic Acids Res.* **2025**, *53*, gkaf942.
47. Sidiqi, M.; Wilce, J. A.; Vivian, J. P.; Porter, C. J.; Barker, A.; Leedman, P. J.; Wilce, M. C. J. Structure and RNA binding of the third KH domain of poly(C)-binding protein 1. *Nucleic Acids Research*, **2005**, *33*, 1213–1221.
48. Ghanem, L. R.; Chatterji, P.; Liebhaber, S. A. Specific enrichment of the RNA-binding proteins PCBP1 and PCBP2 in chief cells of the murine gastric mucosa. *Gene Expr. Patterns*, **2014**, *14*, 78-87.
49. Beckham, S. A.; Matak, M. Y.; Belousoff, M. J.; Venugopal, H.; Shah, N.; Vankadari, N.; Elmlund, H.; Nguyen, J. H. C; Semler, B. L.; Wilce, M. C. J.; Wilce, J. Structure of the PCBP2/stem-loop IV complex underlying translation initiation mediated by the poliovirus type I IRES, *Nucleic Acids Res.* **2020**, *48*, 8006-8021.
50. Vascotto, C.; Fantini, D.; Romanello, M.; Cesaratto, L.; Deganuto, M.; Leonardi, A.; Radicella, J. P.; Kelley, M. r.; D'Ambrosio, C.; Scaloni, A.; Quadrfoglio, F.; Tell, G. APE1/Ref-1 Interacts with NPM1 within nucleoli and plays a role in the rRNA quality control process. *Mol. Cell. Biol*. **2009**, *29*, 1834-1854.
51. Bellina, A.; Malfatti, M. C.; Salgado, G.; Fleming, A. M.; Antoniali, G.; Othman, Z.; Gualandi, N.; La Manna, S.; Marasco, D.; Dassi, E.; Burrows, C.J.; Apurinic/Apyrimidinic Endodeoxyribonuclease 1 modulates RNA G-quadruplex folding of miR-92b and controls its expression in cancer cells. *Proc. Natl. Acad. Sci. U. S. A*. **2024**, *121*, e2317861121.
52. He, H.; Chen, Q.; Georgiadis, M. M. High-Resolution Crystal Structures Reveal Plasticity in the Metal Binding Site of Apurinic/Apyrimidinic Endonuclease I. *Biochemistry*, **2014**, *53*, 6520–6529.
53. Mol, C.; Izumi, T.; Mitra, S.; Tainer, J.A.; DNA-bound structures and mutants reveal abasic DNA binding by APE1 and DNA repair and coordination. *Nature*, **2000**, *403*, 451–456.

54. Kim, E. R.; Selyutina, A. A.; Buldakov, I. A.; Evdokimova, V.; Ovchinnikov, L. P.; Sorokin, A. V. The proteolytic YB-1 fragment interacts with DNA repair machinery and enhances survival during DNA damaging stress. *Cell Cycle*, **2013**, *12*, 3791-3803.
55. Kljashtorny, V.; Nikonov, S.; Ovchinnikov, L.; Kyabin, D.; Vodovar, N.; Curmi, P.; Manivet, P. The cold shock domain of YB-1 segregates RNA from DNA by non-bonded interactions. *PLoS ONE*, **2015**, *10*, e0130318.
56. Tailor, D.; Resendez, A.; Garcia-Marques, F. J.; Pandrala, M.; Going, C. C.; Bermudez, A.; Kumar, V.; Rafat, M.; Nambiar, D. K.; Honkala, A.; Le, Q-T.; Sledge, G. W.; Graves, E.; Pitteri, S. J.; Malhotra, S. V. Y box binding protein 1 inhibition as a targeted therapy for ovarian cancer. *Cell Chem. Biol.* **2021**, *28*, 1206-1220.
57. Ishii, T.; Igawa, T.; Hayakawa, H.; Fujita, T.; Sekiguchi, M.; Nakabeppu, Y. PCB1 and PCB2 both bind heavily oxidized RNA but cause opposing outcomes, suppressing or increasing apoptosis under oxidative conditions. *J. Biol. Chem*. **2020**, *295*, 12247-12261.
